# Pharmacokinetics and drug-drug interaction between enalapril, enalaprilat and felodipine extended release (ER) in healthy subjects

**DOI:** 10.18632/oncotarget.19984

**Published:** 2017-08-07

**Authors:** Dai Li, Sumei Xu, Yulu Wang, Dan Li, Xiaomin Li, Jing Pan, Pingsheng Xu

**Affiliations:** ^1^ National Institution of Drug Clinical Trial, Xiangya Hospital, Central South University, Changsha 410008, China; ^2^ Department of Pharmacy, Xiangya Hospital, Central South University, Changsha 410008, China

**Keywords:** enalapril, enalaprilat, felodipine, interaction, pharmacokinetics

## Abstract

Since angiotensin-converting enzyme (ACE) inhibitors and calcium antagonists have complimentary mechanisms of action, enalapril, an ACE inhibitor, is used in combination with felodipine, a vascular selective dihydropyridine calcium antagonist, for the treatment of hypertension. The present study was designed to investigate the possible drug-drug interaction between these two agents in Chinese healthy subjects. A randomized, open-label, multiple-dose, 3-treatment, 3-period, 6-sequence cross-over study enrolling 12 healthy subjects (six male and six female subjects) was performed. Plasma pharmacokinetic studies were performed after 5 mg of enalapril and 5 mg of felodipine were administered alone or concomitantly twice per day for six days, and once in the morning of day seven. All 12 healthy subjects (mean [SD] age, 24.3 [2.8] years; body weight, 57.3 [5.7] kg; height, 163.2 [5.2] cm) completed all scheduled pharmacokinetic studies. Geometric mean ratios (with 90% CIs) of AUC_τ,ss_ and C_max,ss_ for enalapril administered concomitantly with felodipine *vs.* enalapril administered alone were 1.025 (0.80-1.25) and 1.065 (0.70-1.43), respectively. Geometric mean ratios (with 90% CIs) of AUC_τ,ss_ and C_max,ss_ for felodipine administered concomitantly with enalapril *vs.* felodipine administered alone were 1.14 (0.97-1.31) and 0.80 (0.65-0.95), respectively. There were no severe or serious drug-related adverse events observed during the study. Our results revealed that the co-administration of enalapril and felodipine affected the pharmacokinetics of felodipine, but not that of enalapril. Although the difference in PK parameters was statistically significant, its clinical significance may be limited, considering safety profile observed in the present study.

## INTRODUCTION

The traditional stepwise standard care of hypertension includes the initial step of selecting a single drug, and if necessary, titrating its dosage upward to reach the treatment goal. Additional drugs may be added only if blood pressure (BP) control cannot be achieved with a single agent [1, 2]. It has increasingly been recognized that the upward dose titration of antihypertensive agents can result in a significant increase in side effects with little additional effect on BP control [3-5]. It has now been advocated that combination therapy with low doses of multiple agents can be an alternative strategy for better achieving BP control with fewer adverse effects [6-9].

Combination therapy for hypertension often utilizes various agents with different mechanisms of action. For instance, enalapril reduces peripheral vascular resistance and BP by inhibiting the renin-angiotensin-aldosterone system *via* the blockade of the angiotensin-converting enzyme (ACE) [10]. Felodipine produces vasodilation by reducing calcium entry *via* L-type calcium channels during smooth muscle cell depolarization. Due to its vascular selectivity, felodipine does not suppress myocardial contractility at clinically administered doses [11]. Peripheral edema is a dose-limiting factor for the use of dihydropyridine calcium antagonists, particularly at higher doses [12]. Furthermore, induced edema is not related to fluid retention, but to arteriolar dilation, resulting in an increase in capillary hydrostatic pressure that causes a fluid shift from circulation into the surrounding tissues. By inducing concomitant vasodilatation, enalapril can reduce capillary pressure and the extravasation of fluid into interstitial spaces [13].

The combination of enalapril and felodipine extended release (ER) effectively lowers BP, and is generally well-tolerated [14, 15], with both efficacy and tolerability being enhanced, compared with their monotherapies. Interestingly, various types of calcium channel blockers exert opposite effects on renin secretion. T-type calcium channel blockers can inhibit renin secretion and renin gene expression *in vivo*, whilst L-type calcium channel blockers act as stimulators of the renin system [16]. In the other word, felodipine increase renin secretion that is blocked by co-administration of an ACE inhibitor (enalapril). In addition, both enalapril and felodipine are converted into metabolites via hepatic metabolism. Among the calcium channel blockers, felodipine is commonly used to control hypertension [17], and is most likely to be administered with enalapril. In order to further investigate whether there were potential pharmacokinetic interactions between these two agents, the present study was designed to evaluate the pharmacokinetics (PK) of enalapril and felodipine ER administered alone or in combination in healthy Chinese volunteers. It was hoped that our results would provide a basis for the combination therapy of these two agents, and explore the strategies of combination therapy of these classes of agents in general.

## RESULTS

### Study subjects

Twelve healthy subjects (six males and six females) were enrolled in the study, and all volunteers completed the study. Demographic data of the subjects were as follows (index, mean [±SD]): age, 24.3 (2.8) years; weight, 57.3 (5.7) kg; height, 163.2 (5.2) cm; BMI, 21.2 (1.4) kg/m^2^ (Table 1). The safety analysis set consisted of all 12 participants. There were nostatisticallydifferences in PK parameters between man and women (monotherapy and combination therapy, *P*>0.05).

**Table 1:** Demographic data of the subjects

Number	Sex	Age(y)	Weight(kg)	Height(cm)	BMI(kg/m^2^)
1	female	25	51	159	20
2	female	29	55	166	20
3	male	21	58	163	22
4	female	19	53	160	21
5	male	24	61	163	23
6	female	24	55	155	23
7	male	22	63	167	23
8	female	23	55	160	21
9	male	25	58	172	20
10	male	28	57	161	22
11	female	25	50	160	20
12	male	27	71	172	24

### Pharmacokinetics

**Figure 1:**
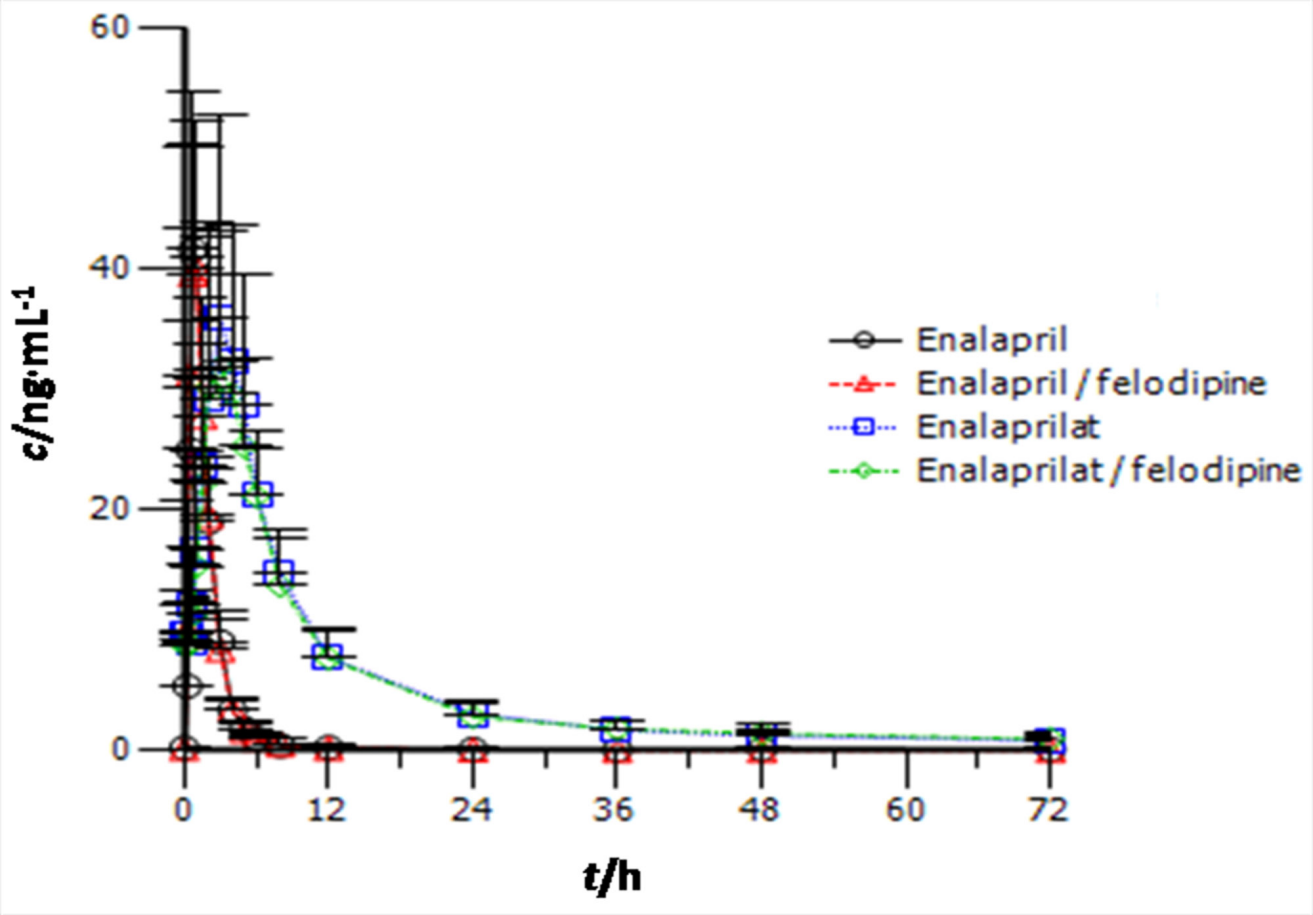
Plasma enalapril and enalaprilat concentration-time profile (mean [±SD]) in healthy subjects after twice-daily administration of enalapril alone and in combination with felodipine

The enalapril plasma concentration-time profile of the co-administration of 5 mg of enalapril with 5 mg of felodipine twice daily for seven days was essentially similar to that with enalapril alone (Figure 1). The calculated PK parameters of enalapril are shown in Table 2. There were no significant differences in C_max,ss_, AUC_τ,ss_, t_1/2_, T_max,ss_, CL/F of enalapril between the enalapril monotherapy, and its combination therapy with felodipine. The estimates (with 90% CIs) of the geometric mean ratios of enalapril C_max,ss_ and AUC_τ,ss_ were 0.99 (0.87-1.14) and 0.97 (0.90-1.05), respectively.

**Table 2:** Pharmacokinetic parameters of enalapril after administration of multiple oral doses of 5 mg of enalapril twice per day (treatment A) and co-administration of 5 mg of enalapril and 5 mg of felodipine twice per day (treatment C) in healthy subjects

Parameter	Enalapril	Enalapril+felodipine	Geometric mean ratio	
	(*n*=12)	(*n*=12)	Point estimate	90% CI
C_max,ss_, ng/mL	44.27 (13.30)	43.15 (8.38)	0.99	0.87-1.14
AUC_τ,ss_, ng.h/mL	84.90 (19.50)	82.70 (16.50)	0.97	0.90-1.05
T_1/2_, h	10.75 (4.59)	11.69 (7.23)		
T_max,ss_, h	0.90 (0.23)	0.92 (0.22)		
CL/F, L/H	64.96 (12.62)	64.19 (12.21)		

The data are expressed as means (±SD).

Enalaprilat plasma concentration-time profile of the co-administration of 5 mg of enalapril with 5 mg of felodipine twice daily for seven days was essentially similar to that with enalapril alone (Figure 1). The calculated PK parameters of enalaprilat are shown in Table 3. There were no significant differences inC_max,ss_, AUC_τ,ss_, t_1/2_, T_max,ss_, CL/F of enalaprilat between the enalapril monotherapy, and its combination therapy with felodipine. The estimates (with 90% CIs) of the geometric mean ratios of the enalaprilat C_max,ss_ and AUC_τ,ss_ were 0.92 (0.79-1.06) and 0.96 (0.85-1.08), respectively.

**Table 3:** Pharmacokinetic parameters of enalaprilat after administration of multiple oral doses of 5 mg of enalapril twice per day (treatment A) and co-administration of 5 mg of enalapril and 5 mg of felodipine twice per day (treatment C) in healthy subjects

Parameter	Enalapril	Enalapril+felodipine	Geometric mean ratio	
	(*n*=12)	(*n*=12)	Point estimate	90% CI
C_max,ss_, ng/mL	37.61 (15.01)	34.07 (11.78)	0.92	0.79-1.06
AUC_τ,ss_, ng.h/mL	372.60 (84.60)	361.00 (90.70)	0.96	0.85-1.08
T_1/2_, h	24.73 (10.29)	27.35 (13.56)		
T_max,ss_, h	3.25 (0.62)	3.17 (0.83)		
CL/F, L/H	22.66 (6.07)	24.12 (6.45)		

The data are expressed as means (±SD).

**Figure 2:**
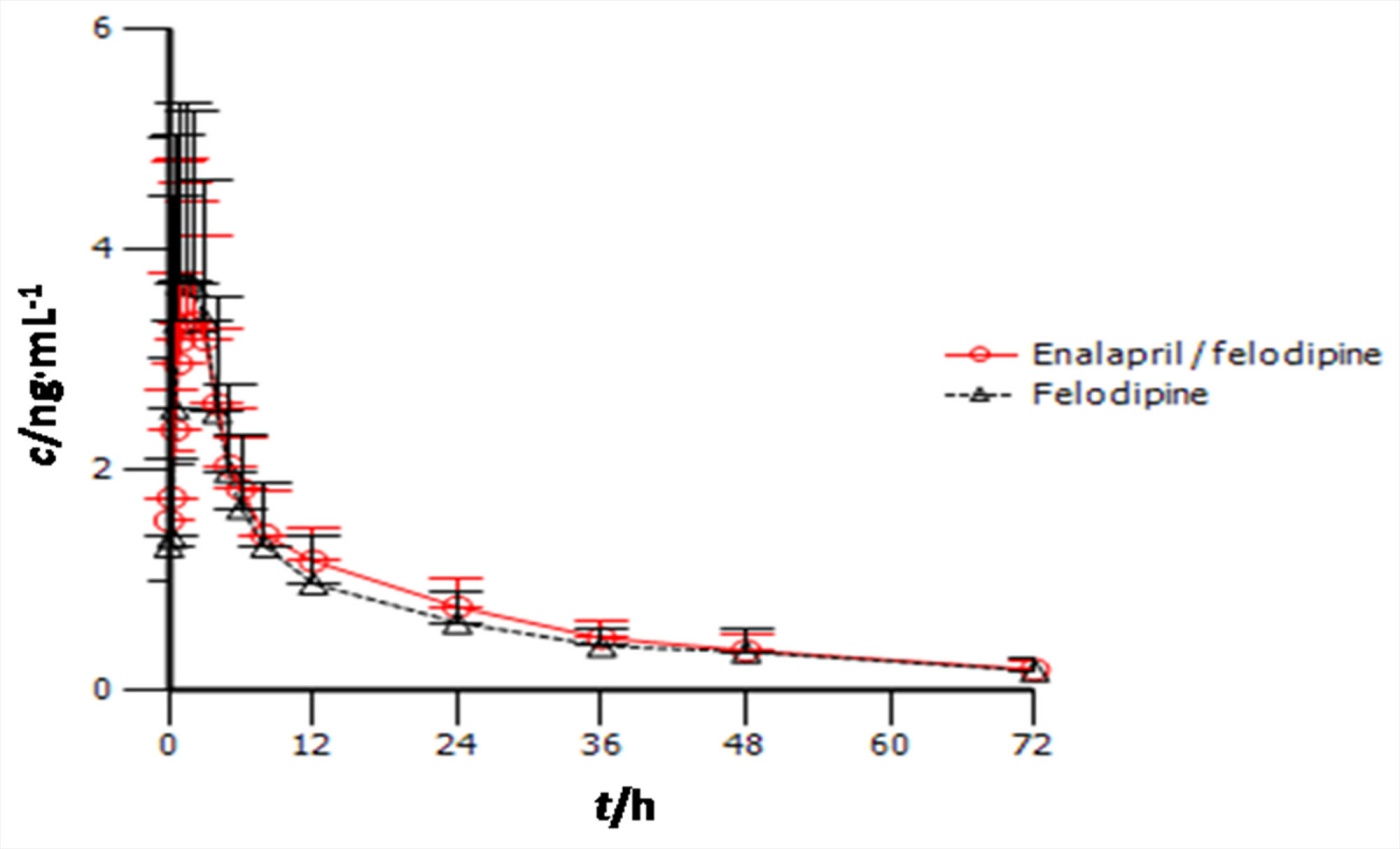
Plasma felodipine concentration-time profile (mean [±SD]) in healthy subjects after twice-daily administration of felodipine alone and in combination with enalapril

The mean plasma concentration-time profiles of felodipine after the administration of 5 mg of felodipine with and without 5 mg of enalapril twice daily for seven days are shown in Figure 2. The estimates (with 90% CIs) of the geometric mean ratios of the felodipine’s C_max,ss_ and AUC_τ,ss_ were 0.79 (0.65-0.95) and 1.13 (0.97-1.30), respectively (Table 4). Although these were statistically significant PK differences, along with a relatively safety profile and regards to, these PK changes do not seem to be of clinical significance.

**Table 4:** Pharmacokinetic parameters of felodipine after oral administration of multiple doses of 5 mg of felodipine twice per day (treatment B) and co-administration of 5 mg of enalapril and 5 mg of felodipine twice per day (treatment C) in healthy subjects

Parameter	Felodipine	Enalapril+felodipine	Geometric mean ratio	
	(*n*=12)	(*n*=12)	Point estimate	90% CI
C_max,ss_, ng/mL	4.82 (1.53)	3.95 (1.70)	0.79	0.65-0.95
AUC_τ,ss_, ng.h/mL	50.60 (20.00)	55.10 (14.10)	1.13	0.97-1.30
T_1/2_, h	25.59 (10.76)	24.15 (8.40)		
T_max,ss_, h	1.73 (0.81)	1.73 (0.86)		
CL/F, L/H	224.15 (58.71)	218.94 (57.91)		

The data are expressed as means (±SD).

### Safety

No serious or severe AEs were reported during the trial. There were 24 AEs (11, 11 and 12 in the enalapril monotherapy, felodipine monotherapy, and combination therapy groups, respectively). All AEs were mild in severity and foreseeable, the participants recovered without sequelae or complication. Among these AEs, 12 subjects had headache, and 12 subjects had dizziness that were considered to be likely related to medicine. In addition, no clinically significant change was seen in the laboratory results, including vital signs, physical examination results, hematology, blood biochemistry, and urinalysis. These results indicate that these two drugs were well-tolerated at the administered doses alone or in combination.

## DISCUSSION

As a long-acting dihydropyridine calcium antagonist, felodipine ER has a highly selective effect on blood vessels; and has been used as an antihypertensive first-line treatment. Enalapril maleate is one of the second generation of ACE inhibitor agents [18], and has long-lasting therapeutic effect and fewer side effects. It is often used for mild to moderate high blood pressure, but its efficacy for severe hypertension remains uncertain, with the increased incidence of side effects at high doses. Therefore, the combination of felodipine and enalapril maleate has several advantages, such as dose reduction for each drug and fewer side effects [19-21]. However, the possibility of an interaction between ACE inhibitor drugs and calcium channel blockers could not be completely ruled out. The present study was designed to evaluate possible PK drug-drug interactions between enalapril and felodipine in Chinese healthy subjects.

Compared with enalapril alone, the C_max,ss_ and AUC_τ,ss_ of enalapril co-administered with felodipine were reduced by 1% and 2%, respectively (Table 1). Compared with enalapril administered alone, the C_max,ss_ and AUC_τ,ss_ of enalaprilat when co-administered with felodipine were reduced by 3% and 11%, respectively (Table 2). There were no significant differences in these PK findings. PK findings of felodipine co-administered with enalapril, compared with felodipine alone, revealed that the AUC_τ,ss_ of felodipine increased by approximately 5% (Table 3). These observations revealed that the PK profiles of enalapril and felodipine in the respective monotherapies were similar to that in the combination therapy.

Enalapril is rapidly absorbed after oral administration with a T_max_ of approximately 0.95 h, and its ethyl part is rapidly hydrolyzed in the liver by hepatic lipase, into an active metabolite enalaprilat. Enalaprilat with a T_max_ of approximately 3.53 h is eliminated with a mean t_1/2_ of approximately 11.32 h [22-23]. Conversely, felodipine is slowly absorbed, with a T_max_ of approximately 2.01 h [24]. The clearance of enalapril to enalprilat is by hepatic CES1 hydrolysis, and the subsequent hydrolysis product, enalaprilat, is eliminated by kindneys. Felodipine is almost completely absorbed after oral administration, and undergoes the first-pass effect in the liver. Therefore, enalapril and felodipine might not influence each other. Statistically, 90% CIs of these PK parameters for enalapril and enalaprilat were within the commonly accepted criteria of 0.8 to 1.25. However, the 90% CIs of C_max,ss_ and AUC_τ,ss_ for felodipine (0.65 to 0.95 and 0.97 to 1.31, respectively) were slightly less than the commonly accepted criteria of 0.8 to 1.25 [25]. Felodipine did not influence the PK of enalapril and enalaprilate, but the PK of felodipine was shown to be affected by enalapril and enalaprilat, with a delay in felodipine elimination. It is worth mentioning that due to the small sample size (n=12), statistical power was <0.8 (the power for C_max_ was just 0.61). The results obtained in the present study should be confirmed in the future large scale studies.

There were no protocol violations or serious adverse events observed in the present study. All adverse events were classified as mild-to-moderate in severity. The incidence rates of adverse drug reactions were similar between enalapril/felodipine alone and enalapril/felodipine in combination with felodipine/enalapril. These results indicated that the combined use of these two drugs is likely to have a similar safety profile as they are administrated alone.

It is noteworthy to mention that since this study was conducted in healthy individuals, the results generated in should be verified in hypertensive patients in the future, especially in patients with liver or renal dysfunctions. The small sample size was a major limitation of the present study and a potential for false positive or false negative findings is possible.

## MATERIALS AND METHODS

### Subjects

Twelve healthy Chinese volunteers (six male and six female) aged 18-40 years with body mass index (BMI) between 19 and 25 kg/m^2^ were enrolled into the present study. Subjects were judged to be eligible for the study when no clinically significant abnormal findings existed on a complete medical examination. The exam included medical history, physical examination, 12-lead electrocardiogram, hematology, blood biochemistry (including renal and hepatic function tests) and urinalysis. Exclusion criteria were as follows: administration of inducers or inhibitors of drug-metabolizing enzymes within one month, a history of allergic disease or diseases that might influence PK parameters of enalapril or felodipine, symptoms of acute disease within four weeks, history of clinically significant hypersensitivity reaction to drugs or foods, systolic blood pressure >140 mm Hg or <90 mm Hg, diastolic blood pressure >90 mm Hg or <50 mm Hg, excessive consumption of caffeine(>5 cups/day), cigarettes(>10 cigarettes/day) or alcohol (30 g alcohol/day), a history of participation in another clinical study within 30 days, a whole-blood donation within 30 days, or positive results on abnormal laboratory test results, including positive results on a urine drug screening or serology tests (hepatitis B surface antigen, anti-hepatitis C virus antibody, and anti-HIV antibody). The subjects were also restricted from those foods (such as grapefruit products) known to affect the absorption, distribution, metabolism, and excretion of the study drugs. These volunteers should have not taken any drugs two weeks prior to the beginning of the study. Furthermore, female subjects should not use contraceptive hormones during the entire study period.

### Study design

This study protocol was reviewed and approved by the Institutional Review Board (IRB) of Xiangya Hospital, Central South University Health System, Changsha, China; and was performed in accordance with the Word Medical Association Declaration of Helsinki, the International Conference on Harmonization Guideline for Human Good Clinical Practice [26]. Each participant was enrolled into the study after providing a written informed consent approved by the IRB.

**Figure 3:**
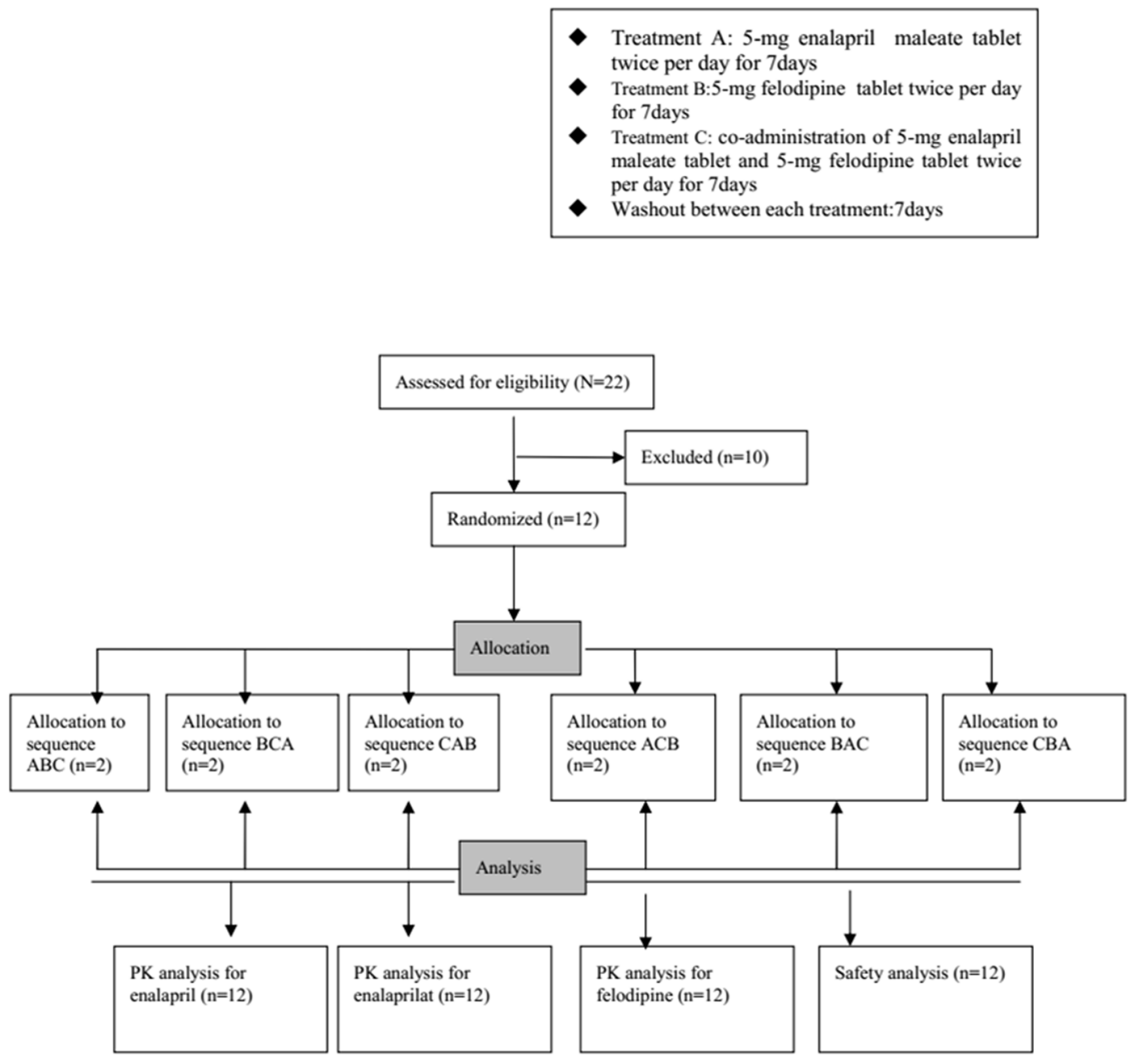
Study design and disposition of subjects

This study used a randomized, open-label, multiple-dose, 3-treatment, 3-period, 6-sequence, two participants per sequence group, male (*n*=6) and female (*n*=6), crossover design. The three types of treatment were as follows: 5-mg enalapril maleate tablet (Hangzhou MSD Pharmaceutical Company Limited; batch number: J001467; specification: 5 mg) twice per day (treatment A), 5-mg felodipine tablet (Jiangsu Lianhuan Pharmaceutical Co., Ltd; batch number: 20121201; specification: 5 mg) twice per day (treatment B), and co-administration of 5-mg enalapril maleate tablet and 5-mg felodipine tablet twice per day (treatment C). The 12 participants were randomly assigned to one of the six treatment-sequence groups (two participants per sequence group). Each sequence consisted of a 7-day cycle of treatments A, B, or C, respectively, in different orders (sequence ABC, BCA, CAB, ACB, BAC, or CBA). Each treatment period was separated by a 7-day washout period (Figure 3).

Each of the three treatments was administered with 250 mL of water under fasting conditions (fasting of more than 10 h) in the morning (8:00 am) and in the evening (20:00 pm) according to the assigned treatment sequence. Peripheral venous blood samples were collected in sodium heparin tubes before dosing on Days 5, 6 and 7 and at 0.25, 0.5, 0.75, 1, 1.5, 2, 3, 4, 5, 6, 8, 12, 24, 36, 48, and 72 h after the last dosing on day seven of each cycle. Blood samples were centrifuged at 3,000 rpm for 10 minutes, and the aliquots of plasma were stored at -80°C until analysis.

### Safety assessment

The safety profile of each subject was included in the safety assessment analysis. All participants were monitored by vital signs (including sitting blood pressure, pulse rate, and body temperature), 12-lead ECG, physical examination, and laboratory tests (including hematology, urinalysis, and blood biochemistry) at indicated time points. In addition, adverse events (AEs) were evaluated by self-reporting or monitoring. The National Cancer Institute Common Toxicity Criteria for Adverse Events version 3.0 was used to describe and grade all toxicities and AEs. The relationship of AEs to the study drug was documented by the investigator as unrelated or unlikely, possibly, probably, or definitely related.

### Bioanalysis

Plasma concentrations of enalapril, enalaprilat and felodipine were measured using a validated high performance liquid chromatography-tandem mass spectrometry(HPLC-MS/MS) assay. A Waters HPLC system (Acquity; Waters, Milford, MA, USA) was coupled with tandem mass spectrometry [27] (API 4000; Applied Biosystems, Foster City, CA, USA). During the optimization of the mass spectrometric parameters, strong and stable signals of analytes were noted and the ion transitions _*m/z*_ 377.4→234.2, _*m/z*_ 349.2→206.0 and _*m/z*_ 384.3→338.4 were selected for the MRM of enalapril, enalaprilat and felodipine, respectively. For felodipine analysis, 500 μL of plasma sample was mixed with 50 μL of nimodipine as an internal standard (2.108 ng/mL) and vortexed for 10 minutes. After centrifugation at 4,000 rpm for 10 minutes, the supernatant (1.4 mL) was collected and evaporated using a nitrogen evaporator (Eyela MG-2200; Tokyo Rikakikai Co, Tokyo, Japan). The residues were reconstituted with 100 μL of HPLC mobile phase, 10 μL of which was injected onto the column at 40°C after centrifugation at 13,000 rpm for five minutes. The mobile phase containing 5 mM of ammonium acetate/acetonitrile (30:70, *vol/vol*) was used at a flow rate of 0.30 mL/min. The lower limit of quantitation was 0.057 ng/mL. The calibration curve was linear over the concentration, which ranged within 0.057 to 20.520 ng/mL (correlation coefficient, *r*^*2*^=0.9972). Intra-day and inter-day precision values were within the range of 3.28% to 6.54% and 3.12% to 8.36%, respectively; and intra-day and inter-day accuracy values were within the range of -6.54% to 3.92% and -0.39% to 4.57%, respectively.

For analyses of enalapril and enalaprilat, solid-phase extraction (SPE) column activation was performed as follows: methanol (1 mL) was added, centrifuged at 1,500 rpm for one minute, and pure water (1 mL) was added; followed by centrifugation at 1,500 rpm for one minute. Then, 500 μL of plasma sample was mixed with 50 μL of benazepril as an internal standard (240.0 ng/mL), 50 μL of mobile phase and 100 μL of phosphoric acid (1M). Then, this was centrifuged at 13,000 rpm for 25 seconds. The supernatant was loaded onto the activated SPE column and centrifuged at 2,500 rpm for two minutes. The column was eluted as follows: (1) 1 mL of 2% formic acid water, and centrifuged at 2,000 rpm for one minute; (2) 0.5 mL of purified water was centrifuged at 2,000 rpm for one minute; (3) after replacement of the collection tube, 1 mL of methanol was added and centrifuged at 2,000 rpm for one minute. The resultant eluent (0.5 mL) was transferred into a 2-mL EP tube, placed in a 40°C water bath, and evaporated under a nitrogen stream. Then, the residue was dissolved in 100 L of mobile phase vortexed for three minutes, centrifuged at 13,000 rpm for three minutes, and 10 μL of the resultant solution was injected directly onto the column. The mobile phase of the methanol/water/formate (70:30:1 [*vol/vol/vol*]) was used at a flow rate of 0.30 mL/min. For enalapril analysis, the lower limit of quantitation was 0.106 ng/mL. The calibration curve was linear over the concentration, ranging within 0.106 to 76.28 ng/mL (*r*^*2*^=0.9962). Intra-day and inter-day precision values of the assay were within the range of 4.70% to 9.05% and 7.74% to 8.69%, respectively; and intra-day and inter-day accuracy values of the assay were within the range of -8.87% to -3.23% and -3.64% to -2.58%, respectively. Forenalaprilat analysis, the lower limit of the quantitation was 0.138 ng/mL. The calibration curve was linear over the concentration, ranging within 0.138 to 99.6 ng/mL (*r*^*2*^=0.9958). Intra-day and inter-day precision values of the assay were within the range of 9.39% to 10.80% and 6.36% to 8.46%, respectively; and intra-day and inter-day accuracy values of the assay were within the range of -0.04% to 5.46% and -0.05% to 8.44%, respectively.

### Pharmacokinetic and statistical analyses

PK parameters of enalapril, enalaprilat and felodipine were calculated using Phoenix 64 WinNonlin 6.3 (Pharsight, Mountain View, CA, USA). AUC_τ,ss_ and C_max,ss_ were estimated after logarithmic transformation. T_max,ss_ was calculated according to non-compartmental analysis, AUC_τ,ss_ was calculated using a linear trapezoidal rule; and k_e_ was estimated by log-linear regression analysis. The values for t_1/2_ and CL/F were calculated using the following equations: t_1/2_ = ln(2) / ke and CL/F = dose/AUC_τ,ss_, respectively. The significance of differences in PK parameters between gender, phase, subject and dosage were determined using analysis of variance (ANOVA).

PK data were analyzed and compared between drug monotherapies (enalapril and felodipine) and the combination therapy. All data were expressed as mean (±SD). Primary PK parameters (C_max,ss_ and AUC_τ,ss_) were log-transformed and analyzed by ANOVA with a mixed-effects model. In order to compare these PK parameters, point estimates and 90% CIs for the geometric mean ratios (combination therapy/monotherapy) of the log-transformed C_max,ss_, and AUC_τ,ss_ were also presented. According to the guidelines of the SFDA of China, the 90% CIs for the geometric mean ratios AUC were within the predetermined range of 0.80 to 1.25 and C_max_ ratios were within 0.70-1.43. Statistical differences between males and females were analyzed using the Kruskal-Wallis test for comparison.

All analyses were conducted using SAS version 9.2 (SAS Institute Inc., Cary, NC, USA). All statistical significance tests were two-sided, and statistical significance was defined as *P*≤0.05.

## Abbreviations

ACE: angiotensin- converting enzyme
AEs: adverse events
BP: blood pressure
BMI: body mass index
ER: extended release
HPLC: high performance liquid chromatography
PK: pharmacokinetics
